# Comparative cost-effectiveness of surgery, angioplasty, or medical therapy in patients with multivessel coronary artery disease: MASS II trial

**DOI:** 10.1186/s12962-018-0158-z

**Published:** 2018-11-03

**Authors:** Sara Michelly Gonçalves Brandão, Paulo Cury Rezende, Hans-Peter Brunner-La Rocca, Yang Ting Ju, Antonio Carlos Pedroso de Lima, Myrthes Emy Takiuti, Whady Hueb, Edimar Alcides Bocchi

**Affiliations:** 1 Instituto do Coração (InCor), Hospital das Clinicas HCFMUSP, Faculdade de Medicina, Universidade de São Paulo, Av Dr Eneas de Carvalho Aguiar 44 – Cerqueira Cesar, São Paulo, SP CEP 05403-000 Brazil; 2 Heart Failure Clinic, Department of Cardiology, Maastricht University Medical Center, Maastricht, The Netherlands; 3 Department of Statistics, Institute of Mathematics and Statistics, University of São Paulo, São Paulo, SP Brazil

**Keywords:** Coronary bypass surgery, Percutaneous coronary intervention, Cost–benefit analysis, Bootstrap method, Bare metal stent

## Abstract

**Background:**

The costs for treating coronary artery disease (CAD) are high worldwide. We performed a prespecified analyses of cost-effectiveness of three therapeutic strategies for multivessel CAD.

**Methods:**

From May 1995 to May 2000, a total of 611 patients were randomly assigned to coronary artery bypass graft (CABG), n = 203; percutaneous coronary intervention (PCI), n = 205; or medical treatment (MT), n = 203. This cost analysis study was based on the perspective of the Public Health Care System. Initial procedural and follow-up costs for medications, cardiology examinations, and hospitalizations for complications were calculated after randomization. Life-years and quality-adjusted life years (QALYs) were used as effectiveness measures. Incremental cost-effectiveness ratios (ICER) were obtained by using nonparametric bootstrapping methods with 5000 resamples.

**Results:**

Initial procedural costs were lower for MT. However, the subsequent 5-year cumulative costs were lower for CABG. Compared with baseline, the three treatment options produced significant improvements in QALYs. After 5 years, PCI and CABG had better QALYs results compared with MT. The ICER results favored CABG and PCI, and favored PCI over CABG in 61% of the drawings. On the other hand, sensitivity analysis showed MT as the preferred therapy compared with CABG and PCI, in the analysis considering higher costs.

**Conclusions:**

At 5-year follow-up, the three treatment options yielded improvements in quality of life, with comparable and acceptable costs. However, despite higher initial costs, the comparison of cost-effectiveness after 5 years of follow-up among the three treatments showed both interventions (CABG and PCI) to be cost-effective strategies compared with MT.

*Trial registration* ISRCTN, ISRCTN66068876, Registered 06/10/1994, http://www.controlled-trials.com/ISRCTN66068876

**Electronic supplementary material:**

The online version of this article (10.1186/s12962-018-0158-z) contains supplementary material, which is available to authorized users.

## Background

Although the therapeutic strategies for patients with stable coronary artery disease and preserved ventricular function have achieved similar outcomes in short- and long-term follow-up, clinical events attributed to these different therapeutic forms are not considered comparatively under the parameters of costs and effectiveness [1, 2]. In this scenario, expectations for additional surgical interventions in patients assigned to clinical or percutaneous treatments have a significant potential for cost increases compared with patients who undergo surgical revascularization as the initial treatment. In addition, the initial cost of medical treatment is usually lower than costs for percutaneous coronary intervention (PCI) and coronary artery bypass graft (CABG), but it is less effective for symptom relief [3]. In addition, in the medium- and long-term, patients may require interventions that increase their costs. Therefore, further need for percutaneous interventions in patients who initially underwent angioplasty may impact changes in cost effectiveness in the long-term follow-up. Considerations regarding the occurrence of major adverse cardiovascular events (MACE) associated with the costs and effectiveness of these strategies may contribute to better decision-making [4]. Thus, this study aimed to analyze and compare the cost-effectiveness of the three therapeutic strategies for multivessel coronary artery disease, medical therapy, bypass surgery, or percutaneous angioplasty in long-term follow-up, in a randomized trial. Furthermore, the cost-effectiveness ratios were also calculated by nonparametric bootstrapping methods with 5000 resamples.

## Methods

### Study design, patient population, and treatment

Details of the Medicine, Angioplasty, or Surgery Study (MASS II) design, study protocol, patient selection, and inclusion criteria have been reported previously [1]. In short, patients with a proximal multivessel coronary stenosis greater than 70% (angiographically documented by visual assessment), as well as documented ischemia, were considered for inclusion. Patients were enrolled and randomized if the surgeons, attending physicians, and interventional cardiologists agreed that revascularization could be attained by either strategy. Clinical criteria for exclusion included refractory angina or acute myocardial infarction (AMI) requiring emergency revascularization and ventricular aneurysm requiring surgical repair. Patients were also excluded if they had valvular heart disease or cardiomyopathy and if they were unable to understand or cooperate with the protocol requirements or return for follow-up.

Patients gave written, informed consent and were randomly assigned to a treatment group. The Ethics Committee of the Hospital das Clínicas da Faculdade de Medicina da University of São Paulo approved the trial under no. 264/94/11, and all procedures were performed in accordance with the Helsinki Declaration.

### Resource utilization and estimation of medical care cost

Cost analysis was performed from the third party payer perspective, i.e., the Brazilian Public Health System, based on reimbursement made by the Ministry of Health through Sistema Único de Saúde (SUS) to the hospital. American Dollar was considered as currency and converted in 2017. The authors only included direct medical costs relevant to the estimated healthcare service using a “top-down” micro-costing approach [5].

The data analyzed included outpatient visits, hospitalization for initial and subsequent revascularization procedures (PCI and CABG), hospitalization that did not involve a revascularization procedure (unstable angina, stroke, or myocardial infarction) as a primary diagnosis of hospitalization, outpatient laboratory tests (glucose, triglycerides, total and fractions of cholesterol in blood levels), outpatient cardiovascular tests [electrocardiogram (ECG), treadmill exercise test (TET), echocardiogram, single photon emission computed tomography (SPE), coronary arteriography (CA)], and outpatient cardiovascular medications (beta-blockers, calcium channel blockers, angiotensin-converting enzyme inhibitors, nitrates, statins, aspirin, oral glucose-lowering agents, and insulin).

### Effectiveness estimation

Life-years gained (LY) and quality-adjusted life-years gained (QALYs) were used as effectiveness measures for the interventions. Life-years were based on overall survival during the 5-year study period. The detailed description of the design and methods for utility weights and QALY beyond the observed 5-year timeframe is provided in Additional file 1: Tables S1–S3 and “Methods”. Briefly, the Quality of Life-Medical Outcomes Study 36-Item Short-Form Health Survey (SF-36) Version 1 (SF-36v1), a generic questionnaire, was used to assess quality of life [6]. The interviews were administered at baseline and at 6, 12, 24, 36, 48, and 60 months of follow-up. To obtain the health state utilities and to measure the QALY, the SF-6D algorithm was applied to the SF-36v1 data collected for this sample [7].

### Cost-effectiveness analyses and threshold

Cost-effectiveness is expressed as incremental cost-effectiveness ratios (ICERs), where incremental costs per LY and QALYs were calculated. The primary analysis is based on QALYs as a preference-based metric for the acceptance of a given health intervention that incorporates the concepts of quality and quantity of life.

No official ICER threshold is applied in Brazil, and the World Health Organization (WHO) recently eliminated the recommendation of using the threshold of three times the per capita gross domestic product (GDP) for averting one QALY [8]. As a result, we arbitrarily defined an intervention “very cost-effective” if it was less than half the gross domestic product (GDP) per capita, “cost-effective” if it was from half to three times the GDP per capita, and “not cost-effective” if it was higher than three times the GDP per capita. The sensitivity analysis data are presented as three times the GDP per capita and $100,000. According to the Instituto Brasileiro de Geografia e Estatística (Brazilian Institute of Geography and Statistics—IBGE), the 2016 *per capita* GDP value was R$ 30,407 (US$ 8783) [9].

Subgroup analyses were performed according to age, sex, diabetes mellitus, Canadian Cardiovascular Society (CCS), angina class, and number of stenosed coronary arteries.

### Sensitivity analysis

We conducted a probability sensitivity analysis (PSA) on the following parameters: cumulative cost, LY, and QALYs for each treatment group. Results of the PSA are presented as cost-effectiveness plane and cost-effectiveness acceptability curves (CEAC).

Costs were increased 26 times for PCI, 15 times for MT, and 26 times for CABG, according to previous studies [10, 11] to make an approach possible for world practice and national market prices, which are referred to as “higher cost” in the text.

In another scenario analysis, called “additional cost,” we considered values that are not included in the imbursement package. In this case, costs of procedures that required more than 2 stents and/or balloons and cardiovascular complications (AMI, unstable angina, stroke) that occurred during hospitalization for revascularization procedures were considered as well.

### Statistical analyses

All data were analyzed according to the intention-to-treat principle. Categorical variables are reported as frequencies and percentages and continuous variables as mean ± standard deviation (SD). χ^2^ test was used to compare qualitative variables among the three groups, and 1-way ANOVA was used for continuous variables. The Kruskal–Wallis tests obtained via bootstrapping with 95% CIs based on 5000 replications were used to assess differences between variables [12]. Significant results for utility and QALY demonstrated by the Kruskal–Wallis test were further analyzed for significance with Dunn’s test. Missing medication data were estimated using the median values for each treatment group, according to each time interval. Missing values for utilities were estimated using multiple imputations adjusted for age, sex, previous myocardial infarction, and diabetes mellitus. Surviving individuals with no information on quality of life were not included (n = 32; PCI = 11, CABG = 15, MT = 6) in cost-effectiveness analysis. Patients who died were censored at the date of death.

Survival data were estimated with the Kaplan–Meier method, and differences among groups were assessed by using the log-rank test. LY at annual time points were estimated as the difference in the area between the Kaplan–Meier survival curves for the two treatment groups [13]. LY and QALYs estimates are reported as means for each group, and differences between groups as 95% confidence intervals (CIs), calculated using bootstrapping (5000 replicates) [12]. Moreover, imbalances in baseline utility values for estimation of mean QALYs in each group were controlled by regression analysis [14]. The covariates included in the model were those that were statistically different among the three treatment groups. Covariates included in the model were: diabetes mellitus, previous MI, smoking status, and angina.

A joint comparison of costs and effects as well as the value of ICER was performed using nonparametric bootstrapping methods with 5000 resamples [12]. The results are presented as an incremental cost-effectiveness plane [15] and as cost-effectiveness acceptability curves (CEAC) [15, 16].

Differences were considered statistically significant when the 95% CIs did not overlap 1.0 or when *P* < 0.05 (2-sided test). All statistical analyses were performed with SPSS 21.0 software or R program version 1.0.136. This study’s reporting followed the 2013 Consolidated Health Economic Evaluation Reporting Standards (CHEERS) guidelines and Good Research Practices for Cost Effectiveness Analysis Alongside Clinical Trials [17, 18].

### Compliance with ethical standards

Financial support for the present study was provided in part by a research grant from the Zerbini Foundation. The Zerbini Foundation also provided for medical writing services. None of the authors has any conflicts of interest to disclose.

## Results

### Patient population

From May 1995 to May 2000, a total of 611 patients with multivessel CAD were randomized to PCI (205), CABG (203), or MT (203). No patient was lost to follow-up. Table 1 summarizes the baseline characteristics of the study patients.

**Table 1:** Baseline characteristics

	PCI (n = 205)	MT (n = 203)	CABG (203)	*P* ^a^
Socio demographic characteristics				
Age, year ± SD	60 ± 9	60 ± 9	60 ± 9	0.959^b^
Female, %	33	31	28	0.412^a^
Clinical characteristics, %				
Hypertension	61	55	63	0.215^a^
Diabetes mellitus	23	36	29	0.062^a^
Previous MI	52	39	41	0.024^a^
Current or past smoker	27	33	32	0.013^a^
CCS angina classes				< 0.001^a^
I and II	72.2	59.1	50.2	
III and IV	20.5	22.6	37.4	
Not applicable or no angina	7.3	18.2	12.3	
Angiographic characteristics, %				
LVEF ± SD	67 ± 8	68 ± 7	67 ± 9	0.984^a^
3-vessels disease	58	59	58	0.980^a^

*MT* medical treatment, *PCI* percutaneous coronary intervention, *CABG* coronary artery bypass graft, *IM* myocardial infarction, *CCS* Canadian Cardiovascular Society angina classes, *LVEF* left ventricular ejection function

^a^Chi square test of homogeneity

^b^ANOVA; SD standard deviation

**Fig. 1:**
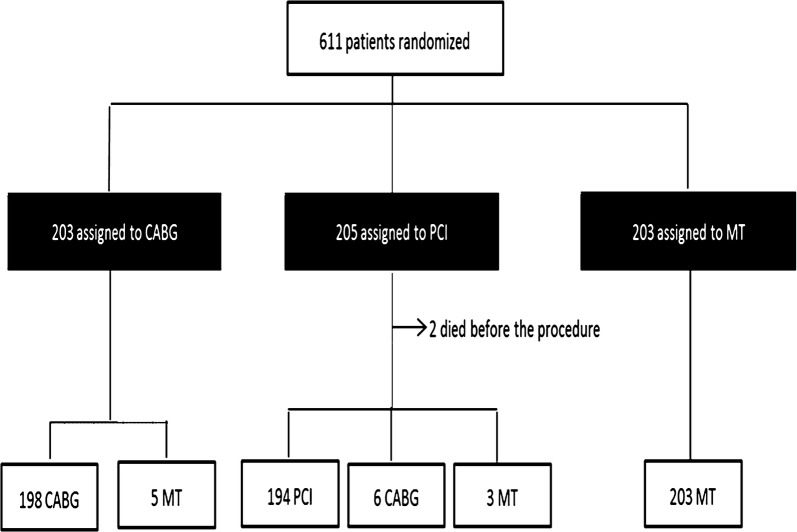
CONSORT (Consolidated Standards of Reporting Trials) diagram. Black boxes represent the intention-to-treat population that was the primary analytic population for the economic study. *PCI* percutaneous coronary intervention, *CABG* coronary artery bypass grafting, *MT* medical treatment

Of the 203 patients assigned to CABG, 198 (98%) received the assigned treatment. However, 5 (2%) received MT because they refused the surgical treatment. Of the 205 patients assigned to the PCI group, 194 (95%) received the assigned treatment, 6 (3%) underwent CABG as their initial treatment, and 2 (0.98%) died before treatment (Fig. 1). Complete revascularization (defined by successful intervention in all major vessels with ≥ 70% stenosis) was achieved in 41% of patients. In addition, 3 patients (1.5%) received MT because they refused the PCI procedure. Among 203 patients assigned to receive MT, 203 (100%) received the assigned treatment. No differences existed among the cumulative overall mortality curves associated with the three therapeutic strategies at the 5-year follow-up (*P *= 0.632) [1].

### Initial events, resource usage and cost

The resources analyzed in this study and the costs per unit are described in Additional file 1: Table S1. During the first 6 months after randomization, clinical events were mostly similar across groups, but AMI was more frequent in the PCI group (15%). Also, the PCI group had a higher incidence of additional PCIs (8.8%). Total index hospitalization costs differed among all groups (*P* < 0.001) and were higher for CABG. Medication costs differed markedly between MT and the other groups (*P* < 0.001). On the other hand, the CABG group had significantly fewer expenses for outpatient care services than the other groups had (*P* < 0.001). Clinical events, procedural resource use, and cost for the initial hospitalization are summarized in Table 2.

**Table 2:** Index events, procedural resource use, and cost

	PCI (n = 205)	MT (n = 203)	CABG (203)	*P*
Procedural resource use, % (n)				
No of PCI procedures				< 0.001^a^
0	6.8 (14)	97.5 (198)	99.5 (202)	
1	96.6 (173)	2.8 (5)	0.6 (1)	
2	8.3 (17)	0.0 (0)	0.0 (0)	
3	0.5 (1)	0.0 (0)	0.0 (0)	
Bare metal stent				< 0.001^a^
0	22.9 (47)	97.5 (198)	100 (203)	
1	35.1 (72)	2.0 (4)	0 (0)	
2	32.2 (66)	0.5 (1)	0 (0)	
3	8.3 (17)	0 (0)	0 (0)	
≥ 4	1.5 (3)	0 (0)	0 (0)	
Balloon angioplasty				< 0.001^a^
0	6.8 (14)	97.5 (198)	99.5 (202)	
1	26.8 (55)	1.5 (3)	0.5 (1)	
2	37.1 (76)	1.0 (2)	0 (0)	
3	21.0 (43)	0 (0)	0 (0)	
≥ 4	8.3 (17)	0 (0)	0 (0)	
CABG procedure				< 0.001^a^
0	92.2 (189)	94.1 (191)	8.9 (18)	
1	7.8 (16)	5.9 (12)	91.1 (185)	
Diagnostic catheterization				< 0.001^a^
1	80.5 (165)	96.6 (196)	95.6 (194)	
2	18.5 (38)	3.4 (7)	4.4 (9)	
3	1.0 (2)	0.0 (0)	0 (0)	
Clinical outcomes, % (n)				
Death	4.9 (10)	1.0 (2)	3.9 (8)	0.07^a^
AMI	14.9 (30)	5.9 (12)	7.4 (15)	0.005^a^
Stroke	2.0 (4)	1.0 (2)	4.4 (9)	0.068^a^
Unstable angina	2.9 (6)	0.5 (1)	1.0 (2)	0.263^a^
Cost, $ mean ± SD				
Medication	47 ± 32 [38]	80 ± 104 [64]	40 ± 29 [30]	< 0.001^b^
Outpatient service	303 ± 85 [274]	284 ± 112 [261]	248 ± 53 [233]	< 0.001^b^
PCI procedure	1490 ± 741 [1385]	35 ± 225 [0]	3 ± 46 [0]	< 0.001^b^
CABG procedure	152 ± 523 [0]	115 ± 460 [0]	1775 ± 555 [1947]	< 0.001^b^
Total cost	1952 ± 841 [1807]	446 ± 525 [277]	2037 ± 559 [2180]	< 0.001^b^

Values in brackets represent medians

*MT* medical treatment, *PCI* percutaneous coronary intervention, *CABG* coronary artery bypass graft, *AMI* acute myocardial infarction, *$* American dollar

^a^Chi square test of homogeneity

^b^Kruskal–Wallis; SD standard deviation

### Follow-up events, resource usage, and costs

**Fig. 2:**
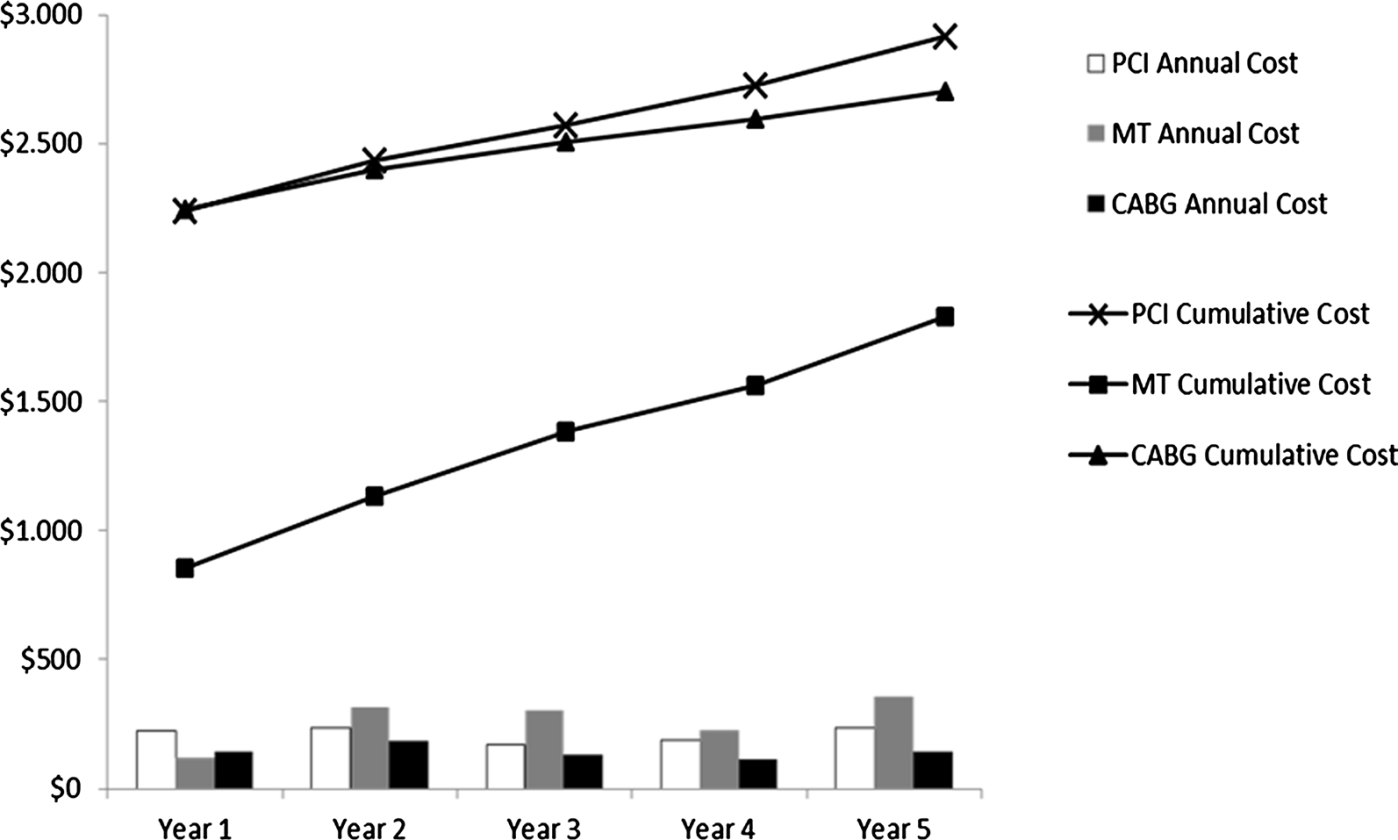
Mean cumulative medical costs (lines) and mean annual follow-up costs (bars) for the percutaneous coronary intervention (PCI), coronary artery bypass grafting (CABG), and medical treatment (MT) groups. Note that the first set of bars represents the costs of the index hospitalization

Over the first year of follow-up, rates of repeat revascularization were higher among patients assigned to initial PCI. During the subsequent year of follow-up, costs for outpatient medications and services were greater for the MT group. The 5-year cumulative medical care costs were notably smaller for the CABG compared with the PCI group (*P* = 0.009) and the MT group (*P* < 0.001), probably because of low rates of repeat revascularization. The index cost of the CABG group had a significant impact on the average long-term cost. Clinical events, resource usage, and costs after the first 6 months are summarized in Table 3. Figure 2 shows the mean annual and cumulative costs of the three treatment strategies.

**Table 3:** Follow-up events, procedural resource use, and cost

	Year 1			Year 2			Year 3		
	PCI (n = 195)	MT (n = 201)	CABG (n = 195)	PCI (n = 191)	MT (n = 197)	CABG (n = 191)	PCI (n = 189)	MT (n = 189)	CABG (n = 185)
Clinical outcomes, n (%)									
Death	4 (2.1)	4 (2.0)	4 (2.1)	2 (1.0)	8 (4.1)	6 (3.1)	0 (0)	11 (5.8)	1 (0.5)
AMI	3 (1.5)	6 (3.0)	2 (1.0)	4 (2.1)	9 (4.6)	4 (2.1)	0 (0)	11 (5.8)	0 (0)
Stroke	1 (0.5)	3 (1.5)	3 (1.5)	0 (0)	3 (1.5)	3 (1.0)	1 (0.5)	2 (1.1)	3 (1.6)
Angina	5 (2.6)	1 (0.5)	0 (0)	3 (1.6)	0 (0)	0 (0)	1 (0.5)	1 (0.5)	3 (1.0)
Resource use, n events/100 patients									
Repeat revascularization (any)	12.3	5.0	4.6	5.8	7.1	3.7	1.6	5.3	1.1
PCI procedure	12.3	2.5	0.5	5.2	3.6	0.5	1.1	1.6	0.5
CABG procedure	0.0	2.5	4.1	0.5	3.6	3.1	0.5	3.7	0.5
Bare metal stent	11.3	3.0	1.0	4.2	4.6	0.5	1.1	1.6	0.5
Balloon angioplasty	21.5	3.0	1.5	9.9	5.6	0.5	1.1	1.6	0.5
Diagnostic catheterization	15.4	5.5	15.9	11.0	6.1	4.2	3.7	10.1	1.1
Cost per patient, $									
Medication	47 ± 32 [39]	80 ± 104 [64]	39 ± 29 [30]	93 ± 63 [78]	162 ± 210 [127]	78 ± 58 [60]	93 ± 63 [78]	163 ± 212 [127]	72 ± 53 [56]
Outpatient service	116 ± 82 [91]	111 ± 116 [85]	90 ± 82 [61]	160 ± 99 [124]	200 ± 229 [157]	115 ± 72 [98]	146 ± 81 [123]	209 ± 223 [166]	110 ± 71 [97]
PCI procedure	150 ± 433 [0]	34 ± 221 [0]	10 ± 141 [0]	63 ± 360 [0]	48 ± 290 [0]	6 ± 90 [0]	13 ± 127 [0]	20 ± 155 [0]	7 ± 91 [0]
CABG procedure	0	48 ± 304 [0]	80 ± 387 [0]	10 ± 141 [0]	69 ± 361 [0]	61 ± 340 [0]	10 ± 141 [0]	72 ± 369 [0]	10 ± 143 [0]
Revascularization procedure (any)	150 ± 433 [0]	83 ± 371 [0]	90 ± 410 [0]	73 ± 385 [0]	118 ± 456 [0]	48 ± 294 [0]	23 ± 190 [0]	92 ± 397 [0]	17 ± 234 [0]
Total	221 ± 474 [54]	120 ± 408 [18]	146 ± 417 [16]	233 ± 432 [124]	316 ± 576 [160]	183 ± 365 [100]	171 ± 222 [123]	306 ± 489 [170]	131 ± 281 [97]

	Year 4			Year 5			5-years cumulative			
	PCI (n = 188)	MT (n = 178)	CABG (n = 183)	PCI (n = 182)	MT (n = 170)	CABG (n = 178)	PCI (n = 195)	MT (n = 201)	CABG (n = 195)	*P* value^c^
Clinical outcomes, n (%)										
Death	7 (3.7)	8 (4.5)	6 (3.3)	5 (2.7)	2 (1.2)	7 (3.9)	18 (9.2)	33 (16.4)	24 (12.3)	0.098^a^
AMI	5 (2.6)	2 (1.1)	1 (0.5)	2 (1.1)	2 (1.2)	7 (3.9)	14 (7.2)	30 (14.9)	14 (7.2)	0.022^a^
Stroke	0 (0)	3 (1.7)	2 (1.1)	2 (1.1)	2 (1.2)	0 (0)	4 (2.1)	13 (6.5)	11 (5.6)	0.178^a^
Angina	2 (1.1)	1 (0.6)	1 (0.5)	4 (2.2)	4 (2.4)	6 (3.4)	15 (7.7)	7 (3.5)	10 (5.1)	0.395^a^
Resource use, n events/100 patients										
Repeat revascularization (any)	3.2	1.7	0.0	5.5	7.6	1.1	27.7	24.9	10.3	0.001^a^
PCI procedure	2.1	1.1	0.0	4.4	2.9	1.1	24.6	10.9	2.6	< 0.001^a^
CABG procedure	1.1	0.6	0.0	1.1	4.7	0.0	3.1	13.9	7.7	< 0.001^a^
Bare metal stent	0.5	1.1	0.0	3.3	4.1	1.1	20.0	13.4	3.1	0.014^a^
Balloon angioplasty	2.7	1.1	0.0	4.4	5.3	1.1	39.0	15.4	3.6	< 0.001^a^
Diagnostic catheterization	4.8	3.4	0.5	12.6	14.7	7.3	46.2	36.3	28.2	0.042^a^
Cost per patient, $										
Medication	93 ± 63 [78]	165 ± 218 [127]	77 ± 57 [72]	92 ± 63 [78]	166 ± 222 [127]	77 ± 57 [60]	403 ± 284 [329]	680 ± 939 [558]	334 ± 255 [262]	< 0.001^b^
Outpatient service	148 ± 83 [123]	199 ± 228 [158]	108 ± 63 [96]	162 ± 97 [127]	220 ± 246 [168]	120 ± 76 [100]	707 ± 373 [635]	867 ± 995 [733]	523 ± 306 [470]	< 0.001^b^
PCI procedure	18 ± 126 [0]	14 ± 131 [0]	0	48 ± 297 [0]	42 ± 257 [0]	14 ± 131 [0]	287 ± 717 [0]	149 ± 473 [0]	36 ± 226 [0]	< 0.001^b^
CABG procedure	20 ± 200 [0]	11 ± 146 [0]	0	21 ± 203 [0]	91 ± 413 [0]	0	60 ± 337 [0]	271 ± 676 [0]	151 ± 522 [0]	< 0.001^b^
Revascularization procedure (any)	38 ± 234 [0]	25 ± 195 [0]	0	69 ± 357 [0]	134 ± 508 [0]	14 ± 131 [0]	347 ± 802 [0]	420 ± 849 [0]	167 ± 553 [0]	0.003^b^
Total	189 ± 283 [123]	229 ± 32 [161]	111 ± 65 [97]	235 ± 413 [133]	359 ± 606 [176]	142 ± 171 [103]	1402 ± 1780 [673]	1707 ± 2090[0]	877 ± 1184 [501]	< 0.001^b^

Values in brackets represent medians

*MT* medical treatment, *PCI* percutaneous coronary intervention, *CABG* coronary artery bypass graft, A*MI* acute myocardial infarction, *$* American dollar

^a^Chi square test of homogeneity

^b^Kruskal–Wallis

^c^P value refers to 5-years cumulative

### Utility weights, QALYs, LYs

Utility values based on SF-6D are summarized in Table 4. Utility weights improved significantly for all groups between baseline and the 6-month follow-up (*P *< 0.001, Bonferroni-corrected). During the subsequent years of follow-up, significant differences were observed among MT and the other two groups (*P *< 0.05, Dunn’s test) at all subsequent time intervals, except at 60 months where the difference was just between the PCI and MT groups (0.809 vs. 0.755, respectively; *P *< 0.05, Dunn’s test). Regarding QALYs and LY results (Table 5), by the end of the 5-year follow-up, QALYs was lower for MT than for CABG and PCI, and no statistical difference was observed between PCI and CABG. The mean cumulative QALYs measurements across the 5 study years were 3.80 for the PCI group, 3.54 for the MT group, and 3.77 for the CABG patients (*P *< 0.05). On the other hand, the results of cumulative LYs did not show significant differences among the three groups at 5-year follow-up and were 4.59 for the PCI group, 4.55 for the MT group, and 4.56 for the CABG.

**Table 4:** Utility of treatments

Period (month)	PCI (n = 194) Mean ± SD	Imputation, n (%)	*MT (n *=* 197)* Mean ± SD	Imputation, n (%)	*CABG (n *=* 188)* Mean ± SD	Imputation n, (%)
Baseline	0.77 ± 0.84 [0.76]	0 (0)	0.76 ± 0.72 [0.76]	0 (0)	0.75 ± 0.07 [0.72]	0 (0)
6	0.79 ± 0.16 [0.79]	5 (2.6)	0.78 ± 0.11 [0.78]	3 (1.5)	0.78 ± 0.13 [0.77]	4 (2.2)
12	0.78 ± 0.19 [0.81]	4 (2.2)	0.77 ± 0.15 [0.78]	5 (2.6)	0.78 ± 0.16 [0.77]	6 (3.3)
24	0.76 ± 0.21 [0.80]	4 (2.2)	0.73 ± 0.22 [0.76]	9 (4.9)	0.76 ± 0.18 [0.78]	12 (6.7)
36	0.77 ± 0.21 [0.81]	5 (2.7)	0.69 ± 0.27 [0.76]	11 (6.3)	0.76 ± 0.21 [0.80]	14 (8.6)
48	0.75 ± 0.24 [0.81]	12 (6.7)	0.66 ± 0.30 [0.76]	10 (6.1)	0.74 ± 0.24 [0.79]	13 (7.9)
60	0.72 ± 0.29 [0.81]	8 (4.7)	0.65 ± 0.31 [0.75]	8 (4.9)	0.72 ± 0.28 [0.78]	11 (6.6)

Values in brackets represent medians; 5000 replications

*MT* medical treatment, *PCI* percutaneous coronary intervention, *CABG* coronary artery bypass graft, *CI* confidence interval, *SD* standard deviation

**Table 5:** Cumulative costs, QALYs and life-years 1–5

Time since randomization, years	Cumulative costs, $	Difference costs, $			Cumulative QALYs			Difference QALYs			Cumulative LYs			Difference LYs		
	CABG PCI MT	CABG vs. MT	PCI vs. MT	PCI vs. CABG	CABG	PCI	MT	CABG vs. MT	PCI vs. MT	PCI vs. CABG	CABG	PCI	MT	CABG vs. MT	PCI vs. MT	PCI vs. CABG
1	2213 2208 645	1568	1563	− 5	0.77	0.78	0.77	0.00	0.01	0.01	0.97	0.96	0.99	− 0.02	− 0.03	− 0.01
2	2388 2428 958	1430	1470	40	1.54	1.54	1.51	0.03	0.03	0.00	1.90	1.88	1.94	− 0.04	− 0.06	− 0.02
3	2508 2585 1243	1265	1342	77	2.29	2.31	2.31	− 0.02	0.00	0.02	2.81	2.80	2.85	− 0.04	− 0.05	− 0.01
4	2609 2759 1443	1166	1317	150	3.04	3.07	2.88	0.16	0.19	0.03	3.70	3.72	3.71	− 0.01	0.01	0.02
5	2753 3002 1740	1013	1262	249	3.77	3.80	3.54	0.23	0.26	0.03	4.56	4.59	4.55	0.01	0.04	0.03

5000 replications

*MT* medical treatment, *PCI* percutaneous coronary intervention, *CABG* coronary artery bypass graft, *QALY* quality-adjusted life-year, *LYs* life-years, *$* American dollar

### Cost-effectiveness analyses: overall population

Results from cost-effectiveness analyses are shown in Table 6. PCI versus MT was cost-effective, with 99% of bootstrap replicates falling below a threshold of 3 GDP per capita per QALY. CABG versus MT was cost-effective as well. Regarding PCI versus CABG, PCI dominated in 35% of the drawings, but was cost-effective in 61%. Assessing outcomes in life-years gained, CABG was associated with a gain of 0.01 in life-years and was dominant in 45% compared with MT (Additional file 1: Table S2).

**Table 6:** Cost-effectiveness analyses for base case and sensitivity analyses per QALY

	Cost, $				QALYs				ICER ($/QALYs)	% Dominant	% Dominated	≤ 3 GDP/capita	≤ $100,000
	PCI	MT	Δ (PCI–MT)	Δ (95% CI)	PCI	MT	Δ (PCI–MT)	Δ (95% CI)					
Overall (n = 391)	3002	1740	1262	(994; 1513)	3.80	3.54	0.26	(0.07; 0.45)	4854	0	0	99	99
Overall and additional cost (n = 391)	3131	1764	1366	(1095; 1630)	3.80	3.54	0.26	(0.07; 0.45)	5255	0	1	98	99
Higher cost (n = 391)	78,063	45,253	32,811	(25,837; 39,331)	3.80	3.54	0.26	(0.07; 0.45)	126,197	0	6	0	25

	Cost, $				QALYs				ICER ($/QALYs)	% Dominant	% Dominated	≤ 3 GDP/capita	≤ $100,000
	CABG	MT	Δ (CABG–MT)	Δ (95% CI)	CABG	MT	Δ (CABG–MT)	Δ (95% CI)					
Overall (n = 385)	2753	1740	1013	(761; 1235)	3.77	3.54	0.23	(0.03; 0.42)	4403	0	1	97	98
Overall and additional cost (n = 385)	2772	1764	1008	(757; 1234)	3.77	3.54	0.23	(0.03; 0.42)	4384	0	1	97	98
Higher cost (n = 385)	71,583	4525	26,331	(19,782; 32,111)	3.77	3.54	0.23	(0.03; 0.42)	114,482	0	8	0	36

	Cost, $				QALYs				ICER ($/QALYs)	% Dominant	% Dominated	≤ 3 GDP/capita	≤ $100,00
	PCI	CABG	Δ (PCI–CABG)	Δ (95% CI)	PCI	CABG	Δ (PCI–CABG)	Δ (95% CI)					
Overall (n = 382)	3002	2753	249	(64; 445)	3.80	3.77	0.03	(− 0.14; 0.21)	8308	0	35	61	64
Overall and additional cost (n = 382)	3130	2772	358	(160; 565)	3.80	3.77	0.03	(− 0.14; 0.21)	11,938	0	35	60	64
Higher cost (n = 382)	78,064	71,583	6480	(1672; 11,566)	3.80	3.77	0.03	(− 0.14; 0.21)	216,014	0	43	4	38

5000 replications

*MT* medical treatment, *PCI* percutaneous coronary intervention, *CABG* coronary artery bypass graft, *CI* confidence interval, *QALY* quality-adjusted life-year, *$* American dollar, *GDP* gross domestic product, *Δ* difference

### Cost-effectiveness analyses: subgroup analysis

Results from subgroup analyses are presented in Additional file 1: Table S3. For most subgroups, the results were consistent with those of the overall trial population; however, some results were relatively unstable owing to the small size of the subgroups.

### Sensitivity analysis

In the sensitivity analysis, items that are not counted by the Public Health Care System reimbursement package were included. This analysis showed that PCI versus MT and CABG versus MT remained attractive at an ICER ≤ 3 GDP/capita (98% and 97%, respectively), and PCI continued to be cost-effective compared with CABG (Table 6).

Finally, in the sensitivity analysis that incorporated international costs, the ICER for PCI versus MT increased, and, thus, PCI was no longer considered cost-effective. Similarly, CABG became less cost-effective than MT. In addition, the cost difference between PCI and CABG increased nearly $10,000 for the overall population; PCI was dominated in 43% of the drawings, but was cost-effective in 38% for a threshold less than $100,000 (Table 6). Different results were seen when the analysis was based on life-years (Additional file 1: Table S4).

**Fig. 3:**
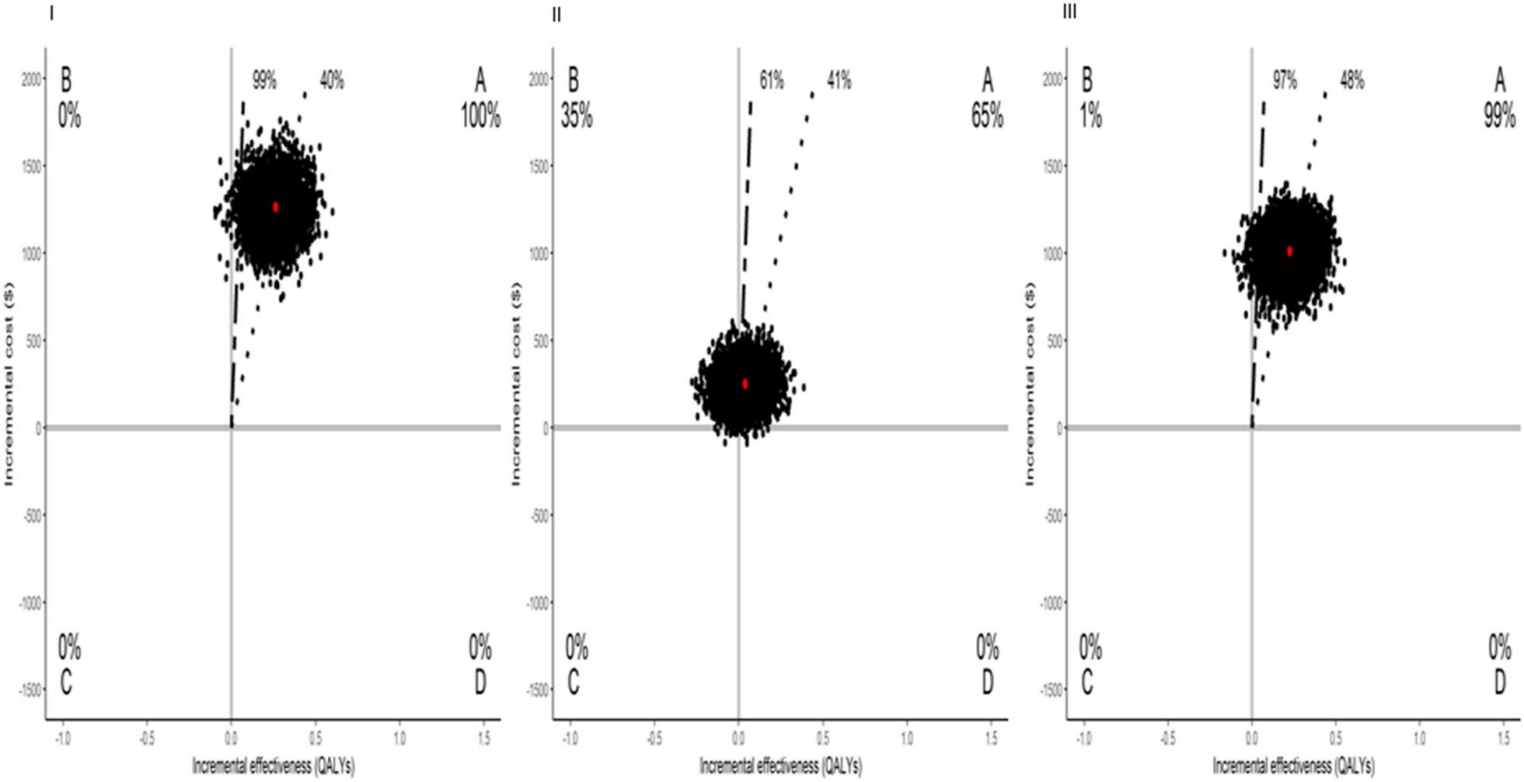
Cost-effectiveness plane in the whole study sample using 5000 replications in bootstrap. Percentages give frequencies of samples in each quadrant. The red circle represents the estimated mean values of our sample. First line (dashed line) represents 0.5 GDP per capita, $4381; second line (medium dashed line) represents 3GDP per capita, $26,288. PCI versus MT (I), PCI versus CABG (II), and CABG versus MT (III). *CABG* coronary artery bypass graft, *PCI* percutaneous coronary intervention, *QALY* quality-adjusted life-year, *$* American dollar, *GDP* gross domestic product

Figure 3 shows a Cost-effectiveness Plane in the whole study sample using 5000 replications after bootstrapping and Additional file 1: Figures S1–S3 shows the Cost-effectiveness acceptability curves.

## Discussion

To the best of our knowledge, this is the first study that analyzes simultaneously cost-effectiveness among three treatment strategies for multivessel CAD in a long-term follow-up. In this analysis, despite substantially higher procedural costs associated with CABG and PCI, differences in costs for MT and revascularization declined across the 5-year follow-up. Moreover, quality of life assessed by QALY showed that revascularization interventions yielded better results compared with MT. Comparing PCI and CABG with each other, QALYs had small differences over 5 years. As a result, both PCI and CABG were cost-effective compared with MT. Nonetheless, PCI versus CABG was modestly cost-effective.

Importantly, compared with baseline data, the three treatment strategies for multivessel CAD yielded improvements in quality of life. Whereas CABG had the highest initial costs, it was associated with fewer clinical events and further revascularizations, fewer medications, and, consequently fewer subsequent long-term costs. PCI had intermediate initial costs, higher need for further revascularizations, but good results in terms of quality of life measures. Finally, MT had lower initial costs, but was associated with higher clinical events and need of medications, and slightly lower quality of life measures after 5 years.

These findings are consistent with previous clinical evidence that suggests that stenting is more cost-effective than off-pump bypass surgery at 1 year. However, it is important to point out that these estimates may lack precision because of sample size, short duration of follow-up, and inclusion of almost 70% of single-vessel disease patients [19]. An economic evaluation of an observational study carried out in London for 6 years compared cost-effectiveness of CABG, PCI, or both revascularization procedures, in 1740 patients. Opposite to our results, in patients suitable for either CABG or PCI treatments, PCI was not cost-effective at a threshold of £30,000 per QALY, and CABG was the most cost-effective form of management (63%). Nevertheless, patients selected for both procedures are not representative of multivessel disease because 41% of them had single-vessel disease and just 8% were triple-vessel disease patients [20]. More recently, a study was carried out by the Iran society perspective to evaluate PCI with stents versus CABG in patients with triple-vessel disease [21]. Contrary to our findings, it found that CABG is a cost-effective strategy compared with PCI, whereas this study included a small and unbalanced sample size, and cardiovascular events were not included in the Markov model over 5 years, 10 years, and life-time horizon. Instead, it used a specific questionnaire to measure QALY, which makes it difficult to compare with other studies [21].

Regarding PCI versus MT, one trial showed that PCI is not cost-effective compared with MT for both in-trial and lifetime extrapolation. Moreover, in-trial results showed PCI to be the dominant strategy in only 19% of the simulations, and that PCI was estimated to cost < $100,000 per QALY gained in only 17% of the patients. Additionally, this trial randomized 34% of single-vessel disease patients [11].

On the other hand, another study that analyzed a decision-analytic model over 5 years with a hypothetical cohort of 10,000 patients with an initial age of 60 years concluded that PCI of chronic total occlusions is cost-effective in patients with severe symptoms compared with optimal medical treatment in patients with chronic stable angina [22].

Some considerations should be pointed out in the present study. This analysis was done based on Public Health Care System reimbursement, and, thus, values for procedures and medications were counted based on a prespecified reimbursement table. In this way, cost differences among individual patients were not counted. On the other hand, the same analytical strategy was done for the three treatment groups, and this analysis balanced such bias. Additionally in percutaneous intervention, only bare metal stents were used to homogenize costs. Considering that this study included CAD patients years ago, the development of new treatment strategies might change our findings. However, this is inherent to long-term follow-up studies. In this sense, studies using off-pump surgery or drug-eluting stents might show different findings. Moreover, this economic analysis was carried out from the Brazilian perspective. To address this issue, costs were also analyzed according to data from previous studies to make a world comparison possible.

## Conclusion

This study shows that the three treatment options for CAD yield improvements in quality of life, with comparable and acceptable costs. However, despite the higher initial costs of CABG and PCI, the comparison of cost-effectiveness after 5 years of follow-up showed both interventions to be cost-effective strategies compared with MT alone.

## Additional file

**Additional file 1.** Additional tables and figures.

## Abbreviations

CA: coronary arteriography
CABG: coronary artery bypass graft
CAD: coronary artery disease
CCS: Canadian Cardiovascular Society
CEAC: cost-effectiveness acceptability curves
CHEERS: Consolidated Health Economic Evaluation Reporting Standards
CIS: confidence intervals
DES: drug-eluting stents
ECG: electrocardiogram
EQ5D: Euroqol 5 Dimensions Questionnaire
GDP: gross domestic product
ICER: incremental cost-effectiveness ratios
LY: life-years gained
MACE: major adverse cardiovascular events
MASS: Medicine, Angioplasty, or Surgery Study
MI: myocardial infarction
MT: medical treatment
PCI: percutaneous coronary intervention
PSA: probability sensitivity analysis
QALY: quality-adjusted life year
QALYs: quality-adjusted life years
SD: standard deviation
SF-36: 36-Item Short-Form Health Survey
SF-36V1: 36-Item Short-Form Health Survey Version 1
SF-6D: Six-Dimensional Health State Classification System
SPE: single photon emission computed tomography
SUS: Sistema Único de Saúde
TET: treadmill exercise test
