# Supplementary material for: Comparative cost-effectiveness of surgery, angioplasty, or medical therapy in patients with multivessel coronary artery disease: MASS II trial
Source: Cost Eff Resour Alloc. 2018 Nov 3;16:55. doi: 10.1186/s12962-018-0158-z (PMC6215652; doi:10.1186/s12962-018-0158-z)
Supplement: Supplementary file 1 — Additional file 1. Additional tables and figures. [file 12962_2018_158_MOESM1_ESM.docx]

**Table S1. Resources analyzed and cost per unit. Brazil, 2017**

| **RESOURCES** | **COST PER UNIT (R$)** | **COST PER UNIT ($)** | **SOURCES** |
| --- | --- | --- | --- |
| EXAMS, PROCEDURES, ESPECIAL MATERIALS |  |  |  |
| Cardiac catheterization | 614.72 | 177.15 | SIGTAP |
| Treadmill exercise test | 30 | 8.65 | SIGTAP |
| Echocardiogram transthoracic | 39.94 | 11.51 | SIGTAP |
| Single photon emission computed tomography | 791.59 | 228.12 | SIGTAP |
| Electrocardiogram | 5.15 | 1.48 | SIGTAP |
| Coronary angioplasty | 1,771.17 | 510.42 | SIGTAP |
| Bare metal stent | 2,034.35 | 586.27 | SIGTAP |
| Baloon Angioplasty | 500 | 144.09 | SIGTAP |
| Coronary artery bypass grafting with extracorporal circulation | 6,756.37 | 1947.08 | SIGTAP |
| HOSPITALIZATION BY CARDIOVASCULAR EVENTS |  |  |  |
| Acute myocardial infarction | 588.12 | 169.49 | SIGTAP |
| Stroke | 463.21 | 133.49 | SIGTAP |
| Unstable angina | 325.08 | 93.68 | SIGTAP |
| OUTPATIENT LABORATORY TESTS |  |  |  |
| Total cholesterol blood levels | 1.85 | 0.53 | SIGTAP |
| High cholesterol blood levels (HDL) | 3.51 | 1.01 | SIGTAP |
| Low cholesterol blood levels (LDL) | 3.51 | 1.01 | SIGTAP |
| Triglycerides | 3.51 | 1.01 | SIGTAP |
| Glucose | 1.85 | 0.53 | SIGTAP |
| OUTPATIENT CARE |  |  |  |
| Outpatients visits | 10 | 2.88 | SIGTAP |
| Beta blocker | 0.22 | 0.06 | BPS |
| Calcium channel blocker | 0.67 | 0.19 | BPS |
| Angiotensin-converting enzyme | 0.37 | 0.11 | BPS |
| Nitrate | 7.20 | 2.07 | BPS |
| Statin | 0.80 | 0.23 | BPS |
| Aspirin | 0.15 | 0.04 | BPS |
| Oral glucose-lowering agents | 0.34 | 0.10 | BPS |
| Insulin | 45.44 | 13.10 | BPS |

BPS, Healthcare Price Bank; SIGTAP, SUS Price Lists and Procedure Management System; R$, Brazilian currency; $, United States Dollars.

# Table S2. Cost-effectiveness analyses for base case and subgroups per QALY

|  | **Cost, $** | | | | | **QALYs** | | | | | **ICER ($/QALY)** | | **% Dominant** | | **% Dominated** | **≤ 0.5 GDP/ capita** | **≤ 3 GDP/ capita** |
| --- | --- | --- | --- | --- | --- | --- | --- | --- | --- | --- | --- | --- | --- | --- | --- | --- | --- |
|  | **PCI** | **MT** | **Δ (PCI - MT)** | **Δ (95% CI)** | | **PCI** | | **MT** | **Δ (PCI - MT)** | **Δ (95% CI)** |  |  |  |  |  |  |  |
| Overall (n = 391) | 3002 | 1740 | 1261 | (994; 1513) | | 3.80 | | 3.54 | 0.26 | (0.07; 0.45) | 4854 | | 0 | | 0 | 40 | 99 |
| Male (n = 267) | 2911 | 1710 | 1201 | (822; 1524) | | 3.85 | | 3.57 | 0.28 | (0.03; 0.54) | 4291 | | 0 | | 2 | 53 | 98 |
| Female (n = 124) | 3197 | 1808 | 1390 | (953; 1843) | | 3.70 | | 3.48 | 0.22 | (-0.05; 0.51) | 6317 | | 0 | | 5 | 25 | 90 |
| Age ≤ 60 (n = 204) | 2946 | 1710 | 1236 | (946; 1540) | | 3.94 | | 3.77 | 0.17 | (-0.04; 0.37) | 7273 | | 0 | | 5 | 14 | 89 |
| Age > 60 (n = 187) | 3064 | 1780 | 1285 | (799; 1685) | | 3.66 | | 3.27 | 0.39 | (0.05; 0.74) | 3294 | | 0 | | 1 | 71 | 98 |
| Diabetes (n = 130) | 3315 | 2088 | 1226 | (593; 1827) | | 3.82 | | 3.29 | 0.53 | (0.17; 0.88) | 2315 | | 0 | | 0 | 91 | 100 |
| No diabetes (n = 261) | 2891 | 1509 | 1382 | (1134; 1629) | | 3.79 | | 3.71 | 0.08 | (-0.14; 0.31) | 17276 | | 0 | | 22 | 3 | 61 |
| Double-vessel disease (n = 182) | 2782 | 1888 | 894 | (541; 1242) | | 3.83 | | 3.65 | 0.18 | (-0.09; 0.45) | 4968 | | 0 | | 10 | 42 | 85 |
| Triple-vessel disease (n = 209) | 3241 | 1637 | 1604 | (1203; 1947) | | 3.78 | | 3.46 | 0.32 | (0.04; 0.58) | 5013 | | 0 | | 1 | 37 | 97 |
| CCS I&II (n = 259) | 2989 | 1783 | 1206 | (861; 1516) | | 3.69 | | 3.53 | 0.16 | (-0.10; 0.41) | 7536 | | 0 | | 12 | 19 | 80 |
| CCS III&IV (n = 81) | 3178 | 1918 | 1260 | (738; 1773) | | 4.00 | | 3.62 | 0.38 | (0.15; 0.64) | 3317 | | 0 | | 0 | 75 | 100 |
|  | **CABG** | **MT** | **Δ (CABG - MT)** | **Δ (95% CI)** | | **CABG** | | **MT** | **Δ (CABG - MT)** | **Δ (95% CI)** | **ICER ($/QALY)** | | **% Dominant** | | **% Dominated** | **≤ 0.5 GDP/ capita** | **≤ 3 GDP/ capita** |
| Overall (n = 385) | 2753 | 1740 | 1012 | (761; 1235) | | 3.77 | | 3.54 | 0.23 | (0.03; 0.42) | 4403 | | 0 | | 1 | 48 | 97 |
| Male (n = 273) | 2700 | 1710 | 990 | (671; 1255) | | 3.83 | | 3.57 | 0.26 | (0.05; 0.49) | 3809 | | 0 | | 1 | 63 | 98 |
| Female (n = 112) | 2900 | 1808 | 1092 | (725; 1477) | | 3.58 | | 3.48 | 0.10 | (-0.20; 0.39) | 10919 | | 0 | | 24 | 17 | 67 |
| Age ≤ 60 (n = 205) | 2799 | 1710 | 1089 | (849; 1317) | | 3.82 | | 3.77 | 0.05 | (-0.19; 0.28) | 21784 | | 0 | | 34 | 5 | 53 |
| Age > 60 (n = 180) | 2702 | 1780 | 923 | (473; 1283) | | 3.70 | | 3.27 | 0.43 | (0.13; 0.75) | 2146 | | 0 | | 0 | 92 | 100 |
| Diabetes (n = 147) | 2821 | 2088 | 733 | (228; 1143) | | 3.68 | | 3.29 | 0.39 | (0.03; 0.74) | 1879 | | 0 | | 1 | 88 | 97 |
| No diabetes (n = 238) | 2714 | 1509 | 1205 | (992; 1414) | | 3.81 | | 3.71 | 0.10 | (-0.09; 0.30) | 12050 | | 0 | | 15 | 6 | 71 |
| Double-vessel disease (n = 162) | 2771 | 1888 | 883 | (604; 1149) | | 3.89 | | 3.65 | 0.24 | (0.02; 0.48) | 3680 | | 0 | | 1 | 64 | 97 |
| Triple-vessel disease (n = 223) | 2739 | 1637 | 1102 | (745; 1385) | | 3.67 | | 3.46 | 0.21 | (-0.08; 0.50) | 5248 | | 0 | | 8 | 39 | 87 |
| CCS I&II (n = 211) | 2755 | 1783 | 972 | (587; 1284) | | 3.79 | | 3.53 | 0.26 | (0.00; 0.51) | 3737 | | 0 | | 3 | 59 | 95 |
| CCS III&IV (n = 114) | 2790 | 1918 | 872 | (437; 1238) | | 3.71 | | 3.62 | 0.09 | (-0.22; 0.41) | 9691 | | 0 | | 29 | 25 | 64 |
|  | **PCI** | **CABG** | **Δ (PCI -CABG)** | **Δ (95% CI)** | | **PCI** | | **CABG** | **Δ (PCI -CABG)** | **Δ (95% CI)** | **ICER ($/QALY)** | | **% Dominant** | | **% Dominated** | **≤ 0.5 GDP/ capita** | **≤ 3 GDP/ capita** |
| Overall (n = 382) | 3002 | 2753 | 249 | (64; 445) | | 3.80 | | 3.77 | 0.03 | (-0.14; 0.21) | 8308 | | 0 | | 35 | 41 | 61 |
| Male (n = 268) | 2911 | 2700 | 211 | (-2; 436) | | 3.85 | | 3.83 | 0.02 | (-0.22; 0.25) | 10553 | | 1 | | 42 | 40 | 53 |
| Female (n = 114) | 3197 | 2900 | 298 | (-85; 685) | | 3.70 | | 3.58 | 0.12 | (-0.18; 0.44) | 2482 | | 5 | | 21 | 57 | 71 |
| Age ≤ 60 (n = 197) | 2946 | 2799 | 147 | (-119; 413) | | 3.94 | | 3.82 | 0.12 | (-0.10; 0.34) | 1226 | | 12 | | 13 | 64 | 72 |
| Age > 60 (n = 185) | 3064 | 2702 | 362 | (103; 618) | | 3.66 | | 3.70 | -0.04 | (-0.30;0.23) | -9047 | | 0 | | 61 | 18 | 35 |
| Diabetes (n = 119) | 3315 | 2821 | 494 | (84; 941) | | 3.82 | | 3.68 | 0.14 | (-0.16; 0.46) | 3527 | | 1 | | 18 | 57 | 77 |
| Table S2. Cost-effectiveness analyses for base case and subgroups per QALY (continued) | | | | | | | | | | | | | | | | | |
|  | **PCI** | **CABG** | **Δ (PCI -CABG)** | **Δ (95% CI)** | | **PCI** | | **CABG** | **Δ (PCI -CABG)** | **Δ (95% CI)** | | **ICER ($/QALY)** | | **% Dominant** | **% Dominated** | **≤ 0.5 GDP/ capita** | **≤ 3 GDP/ capita** |
| No diabetes (n = 263) | 2891 | 2714 | 177 | (-24; 378) | | 3.79 | | 3.81 | -0.02 | (-0.23; 0.19) | | -8856 | | 2 | 52 | 28 | 40 |
| Double-vessel disease (n = 180) | 2782 | 2771 | 11 | (-223; 252) | | 3.83 | | 3.89 | -0.06 | (-0.28; 0.13) | | -182 | | 10 | 36 | 12 | 16 |
| Triple-vessel disease (n = 202) | 3241 | 2739 | 502 | (245; 770) | 3.78 | | 3.67 | | 0.11 | (-0.19; 0.39) | | 4565 | | 0 | 23 | 48 | 73 |
| CCS I&II (n = 234) | 2989 | 2755 | 234 | (-10; 477) | 3.69 | | 3.79 | | -0.10 | (-0.34; 0.14) | | -2342 | | 0 | 77 | 10 | 18 |
| CCS III&IV (n = 109) | 3178 | 2790 | 388 | (27; 800) | 4.00 | | 3.71 | | 0.29 | (0.03; 0.57) | | 1338 | | 2 | 1 | 90 | 96 |

# MT, medical treatment; PCI, percutaneous coronary intervention; CABG, coronary artery bypass graft; CI, confidence interval; QALY, quality-adjusted life-year; CCS, Canadian Cardiovascular Society angina classes; $, American dollar; GDP, gross domestic product; Δ = difference, 5000 replications.

# Table S3. Cost-effectiveness analyses for base case and sensitivity analyses per LYs

|  | **Cost, $** | | | | **LYs** | | | | | **ICER ($/LYs)** | **% Dominant** | **% Dominated** | **≤ 3 GDP/ capita** | **≤ $100.000** |
| --- | --- | --- | --- | --- | --- | --- | --- | --- | --- | --- | --- | --- | --- | --- |
|  | **PCI** | **MT** | **Δ (PCI - MT)** | **Δ (95% CI)** | **PCI** | **MT** | **Δ (PCI - MT)** | | **Δ (95% CI)** |  |  |  |  |  |
| Overall (n = 408) | 2967 | 1744 | 1223 | (958; 1462) | 4.60 | 4.55 | 0.05 | (-0.18; 0.27) | | 24458 | 0 | 34 | 51 | 63 |
| Overall and additional cost (n = 408) | 3104 | 1768 | 1336 | (1058; 1585) | 4.60 | 4.55 | 0.05 | (-0.18; 0.27) | | 26725 | 0 | 35 | 50 | 63 |
| Higher cost (n = 408) | 77147 | 45351 | 31796 | (24905; 37999) | 4.60 | 4.55 | 0.05 | (-0.18; 0.27) | | 635918 | 0 | 70 | 0 | 1 |
|  | **CABG** | **MT** | **Δ (CABG - MT)** | **Δ (95% CI)** | **CABG** | **MT** | **Δ (CABG - MT)** | **Δ (95% CI)** | | **ICER ($/LYs)** | **% Dominant** | **% Dominated** | **≤ 3 GDP/ capita** | **≤ $100.000** |
| Overall (n = 406) | 2734 | 1744 | 989 | (752; 1207) | 4.56 | 4.55 | 0.01 | (-0.22; 0.24) | | 98946 | 0 | 45 | 43 | 52 |
| Overall and additional cost (n = 406) | 2752 | 1768 | 984 | (742; 1204) | 4.56 | 4.55 | 0.01 | (-0.22; 0.24) | | 98406 | 0 | 45 | 43 | 52 |
| Higher cost (n = 406) | 71077 | 45351 | 25726 | (19558; 31381) | 4.56 | 4.55 | 0.01 | (-0.22; 0.24) | | 2572588 | 0 | 74 | 0 | 2 |
|  | **PCI** | **CABG** | **Δ (PCI -CABG)** | **Δ (95% CI)** | **PCI** | **CABG** | **Δ (PCI -CABG)** | **Δ (95% CI)** | | **ICER ($/LYs)** | **% Dominant** | **% Dominated** | **≤ 3 GDP/ capita** | **≤ $100.000** |
| Overall (n = 408) | 2967 | 2734 | 233 | (61; 411) | 4.60 | 4.56 | 0.04 | (-0.20; 0.26) | | 5836 | 0 | 39 | 58 | 60 |
| Overall and additional cost (n = 408) | 3104 | 2752 | 352 | (166; 547) | 4.60 | 4.56 | 0.04 | (-0.20; 0.26) | | 8805 | 0 | 39 | 57 | 60 |
| Higher cost (n = 408) | 77147 | 71077 | 6070 | (1578; 10690) | 4.60 | 4.56 | 0.04 | (-0.20; 0.26) | | 151751 | 0 | 45 | 7 | 40 |

# MT, medical treatment; PCI, percutaneous coronary intervention; CABG, coronary artery bypass graft; CI, confidence interval; LYs, life-years; $, American dollar; GDP, gross domestic product; Δ = difference, 5000 replications.

# Table S4. Cost-effectiveness analyses for base case and subgroups per LYs

|  | **Cost, $** | | | | | **LYs** | | | | | **ICER ($/LYs)** | **% Dominant** | **% Dominated** | **≤ 0.5 GDP/per capita** | **≤ 3 GDP/per capita** |
| --- | --- | --- | --- | --- | --- | --- | --- | --- | --- | --- | --- | --- | --- | --- | --- |
|  | **PCI** | | **MT** | **Δ (PCI - MT)** | **Δ (95% CI)** | **PCI** | | **MT** | **Δ (PCI - MT)** | **Δ (95% CI)** |  |  |  |  |  |
| Overall (n = 408) | 2967 | | 1744 | 1223 | (958; 1462) | 4.60 | | 4.55 | 0.05 | (-0.18; 0.27) | 24458 | 0 | 34 | 2 | 51 |
| Male (n = 276) | 2882 | | 1724 | 1158 | (814; 1457) | 4.60 | | 4.50 | 0.10 | (-0.17; 0.37) | 11577 | 0 | 29 | 12 | 66 |
| Female (n = 132) | 3142 | | 1781 | 1366 | (929; 1805) | 4.57 | | 4.64 | -0.07 | (-0.43; 0.28) | -19438 | 0 | 69 | 1 | 27 |
| Age ≤ 60 (n = 213) | 2904 | | 1711 | 1193 | (874; 1504) | 4.81 | | 4.76 | 0.05 | (-0.19; 0.28) | 23866 | 0 | 43 | 3 | 50 |
| Age > 60 (n = 195) | 3032 | | 1780 | 1252 | (788; 1649) | 4.38 | | 4.29 | 0.09 | (-0.30; 0.49) | 13909 | 0 | 37 | 16 | 58 |
| Diabetes (n = 137) | 3289 | | 2077 | 1212 | (620; 1742) | 4.54 | | 4.28 | 0.26 | (-0.22; 0.70) | 4662 | 0 | 17 | 47 | 81 |
| No diabetes (n = 271) | 2850 | | 1515 | 1335 | (1098; 1575) | 4.62 | | 4.73 | -0.11 | (-0.34; 0.11) | -12136 | 0 | 89 | 0 | 8 |
| Double-vessel disease (n = 186) | 2796 | | 1899 | 897 | (544; 1243) | 4.69 | | 4.63 | 0.06 | (-0.25; 0.38) | 14943 | 0 | 39 | 18 | 57 |
| Triple-vessel disease (n = 222) | 3145 | | 1635 | 1510 | (1140; 1848) | 4.49 | | 4.49 | 0.00 | (-0.35; 0.33) | 0 | 0 | 55 | 2 | 39 |
| CCS I&II (n = 268) | 2940 | | 1766 | 1174 | (803; 1505) | 4.51 | | 4.53 | -0.02 | (-0.31; 0.27) | -58715 | 0 | 62 | 2 | 33 |
| CCS III&IV (n = 88) | 3160 | | 1971 | 1189 | (699; 1687) | 4.75 | | 4.74 | 0.01 | (-0.39; 0.37) | 118903 | 0 | 52 | 8 | 44 |
|  | **CABG** | | **MT** | **Δ (CABG - MT)** | **Δ (95% CI)** | **CABG** | **MT** | | **Δ (CABG - MT)** | **Δ (95% CI)** | **ICER ($/LYs)** | **% Dominant** | **% Dominated** | **≤ 0.5 GDP/per capita** | **≤ 3 GDP/per capita** |
| Overall (n = 406) | 2734 | | 1744 | 989 | (752; 1207) | 4.56 | 4.55 | | 0.01 | (-0.22; 0.24) | 98946 | 0 | 45 | 4 | 43 |
| Male (n = 287) | 2680 | | 1724 | 956 | (659; 1207) | 4.56 | 4.50 | | 0.06 | (-0.21; 0.32) | 15928 | 0 | 40 | 12 | 55 |
| Female (n = 119) | 2872 | | 1781 | 1090 | (741; 1457) | 4.57 | 4.64 | | -0.07 | (-0.49; 0.33) | -15575 | 0 | 67 | 6 | 30 |
| Age ≤ 60 (n = 214) | 2777 | | 1711 | 1066 | (828; 1243) | 4.66 | 4.76 | | -0.10 | (-0.35; 0.14) | -10658 | 0 | 84 | 0 | 13 |
| Age > 60 (n = 192) | 2685 | | 1780 | 905 | (466; 1241) | 4.46 | 4.29 | | 0.17 | (-0.23; 0.57) | 5324 | 0 | 23 | 42 | 75 |
| Diabetes (n = 155) | 2801 | | 2077 | 723 | (209; 1127) | 4.51 | 4.28 | | 0.23 | (-0.22; 0.67) | 3145 | 0 | 18 | 61 | 81 |
| No diabetes (n = 251) | 2695 | | 1515 | 1180 | (970; 1389) | 4.59 | 4.73 | | -0.14 | (-0.39; 0.10) | -8426 | 0 | 91 | 0 | 6 |
| Double-vessel disease (n = 168) | 2763 | | 1899 | 864 | (589; 1131) | 4.81 | 4.63 | | 0.18 | (-0.08; 0.46) | 4797 | 0 | 12 | 45 | 86 |
| Triple-vessel disease (n = 238) | 2710 | | 1635 | 1075 | (726; 1359) | 4.38 | 4.49 | | -0.11 | (-0.45; 0.24) | -9777 | 0 | 75 | 2 | 21 |
| CCS I&II (n = 222) | 2730 | | 1766 | 964 | (597; 1273) | 4.60 | 4.53 | | 0.07 | (-0.25; 0.37) | 13771 | 0 | 37 | 18 | 59 |
| CCS III&IV (n = 122) | 2765 | | 1971 | 794 | (402; 1160) | 4.50 | 4.74 | | -0.24 | (-0.62; 0.13) | -3310 | 0 | 92 | 2 | 7 |
|  | **PCI** | | **CABG** | **Δ (PCI -CABG)** | **Δ (95% CI)** | **PCI** | **CABG** | | **Δ (PCI -CABG)** | **Δ (95% CI)** | **ICER ($/LYs)** | **% Dominant** | **% Dominated** | **≤ 0.5 GDP/per capita** | **≤ 3 GDP/per capita** |
| Overall (n = 408) | 2967 | | 2734 | 233 | (61; 411) | 4.60 | 4.56 | | 0.04 | (-0.20; 0.26) | 5837 | 0 | 39 | 43 | 58 |
| Male (n = 283) | 2882 | | 2680 | 202 | (4; 409) | 4.60 | 4.56 | | 0.04 | (-0.22; 0.31) | 202 | 1 | 37 | 49 | 60 |
| Female (n = 125) | 3142 | | 2872 | 270 | (-33; 581) | 4.57 | 4.57 | | 0.00 | (-0.46; 0.48) | 270 | 2 | 48 | 39 | 48 |
| Age ≤ 60 (n = 207) | 2904 | | 2777 | 127 | (-142; 400) | 4.81 | 4.66 | | 0.15 | (-0.10; 0.41) | 127 | 14 | 11 | 67 | 72 |
| Table S4. Cost-effectiveness analyses for base case and subgroups per LYs (continued) | | | | | | | | | | | | | | | |
|  | | **PCI** | **CABG** | **Δ (PCI -CABG)** | **Δ (95% CI)** | **PCI** | **CABG** | | **Δ (PCI -CABG)** | **Δ (95% CI)** | **ICER ($/LYs)** | **% Dominant** | **% Dominated** | **≤ 0.5 GDP/per capita** | **≤ 3 GDP/per capita** |
| Age > 60 (n = 201) | | 3032 | 2685 | 347 | (104; 599) | 4.38 | 4.46 | | -0.08 | (-0.46; 0.31) | 347 | 0 | 66 | 20 | 33 |
| Diabetes (n = 126) | | 3289 | 2801 | 489 | (105; 907) | 4.54 | 4.51 | | 0.03 | (-0.48; 0.51) | 489 | 0 | 45 | 36 | 52 |
| No diabetes (n = 282) | | 2850 | 2695 | 155 | (-36; 348) | 4.62 | 4.59 | | 0.03 | (-0.24; 0.31) | 155 | 3 | 39 | 46 | 55 |
| Double-vessel disease (n = 188) | | 2796 | 2763 | 33 | (-197; 272) | 4.69 | 4.81 | | -0.12 | (-0.35; 0.11) | 33 | 5 | 49 | 8 | 11 |
| Triple-vessel disease (n = 220) | | 3145 | 2710 | 434 | (197; 682) | 4.49 | 4.38 | | 0.11 | (-0.26; 0.48) | 434 | 0 | 30 | 52 | 69 |
| CCS I&II (n = 250) | | 2940 | 2730 | 210 | (-15; 438) | 4.51 | 4.60 | | -0.09 | (-0.39; 0.22) | 210 | 1 | 70 | 18 | 26 |
| CCS III&IV (n = 118) | | 3160 | 2765 | 395 | (63; 777) | 4.75 | 4.50 | | 0.25 | (-0.15; 0.65) | 395 | 1 | 12 | 78 | 87 |

# MT, medical treatment; PCI, percutaneous coronary intervention; CABG, coronary artery bypass graft; CI, confidence interval; LYs, life-years; GDP, CCS, Canadian Cardiovascular Society angina classes; gross domestic product; $, American dollar; Δ = difference, 5000 replications.

# Figure S1. Cost-effectiveness acceptability curves of Percutaneous Coronary Intervention versus Medical Treatment in 3 distinct analysis.

#
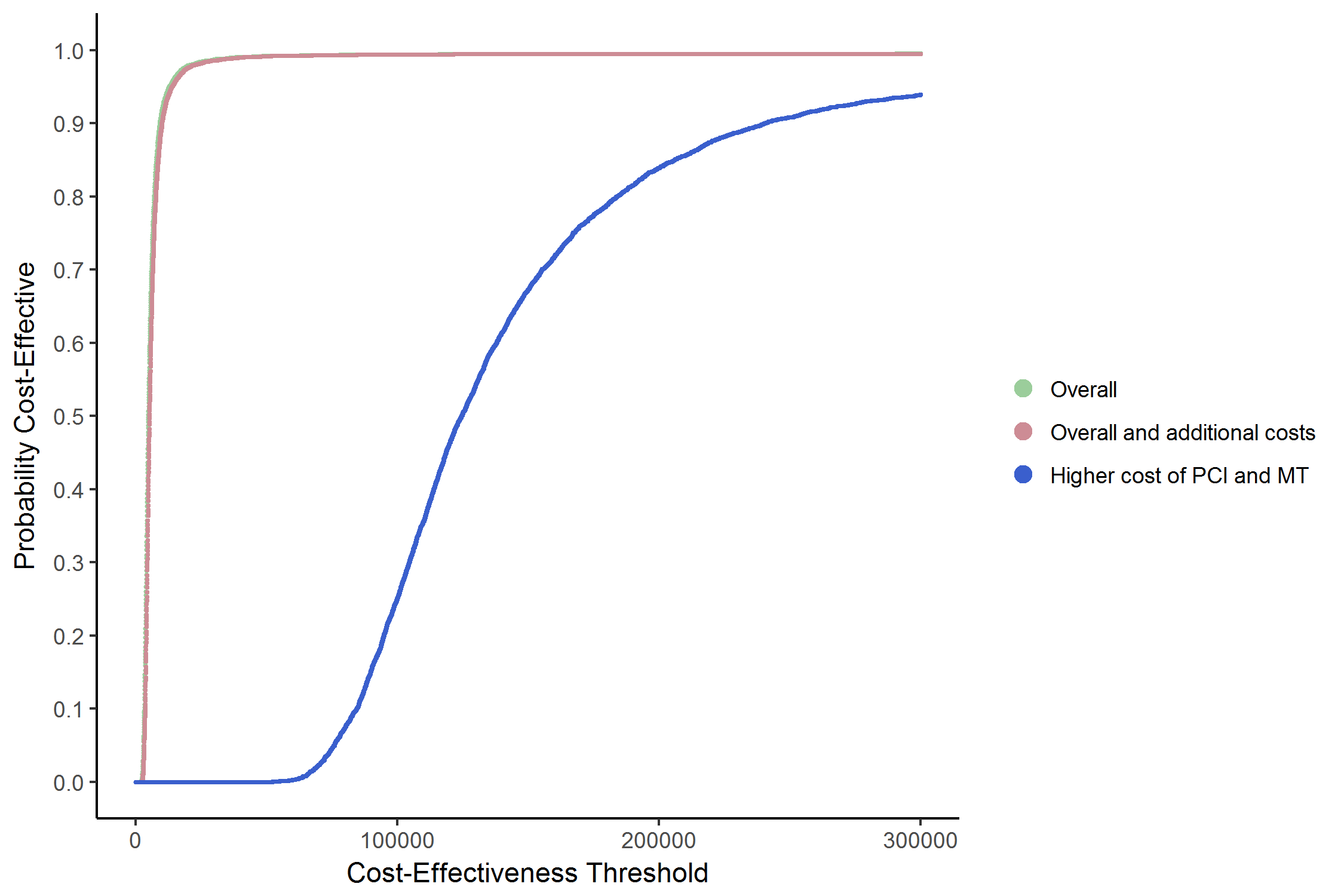


# Footnote: In 2 analysis (Overall, Overall and additional costs), the probability that PCI is cost-effective compared to MT is very high, even at low threshold values. In Higher Cost analysis, the probability that PCI is cost-effective compared to MT increases as cost-effectiveness threshold rises to more than 100,000 US dollars. PCI percutaneous coronary intervention, MT medical treatment, threshold is in US dollars.

Figure S2. Cost-effectiveness acceptability curves of Percutaneous Coronary Intervention versus Coronary Artery Bypass Grafting in 3 distinct analysis


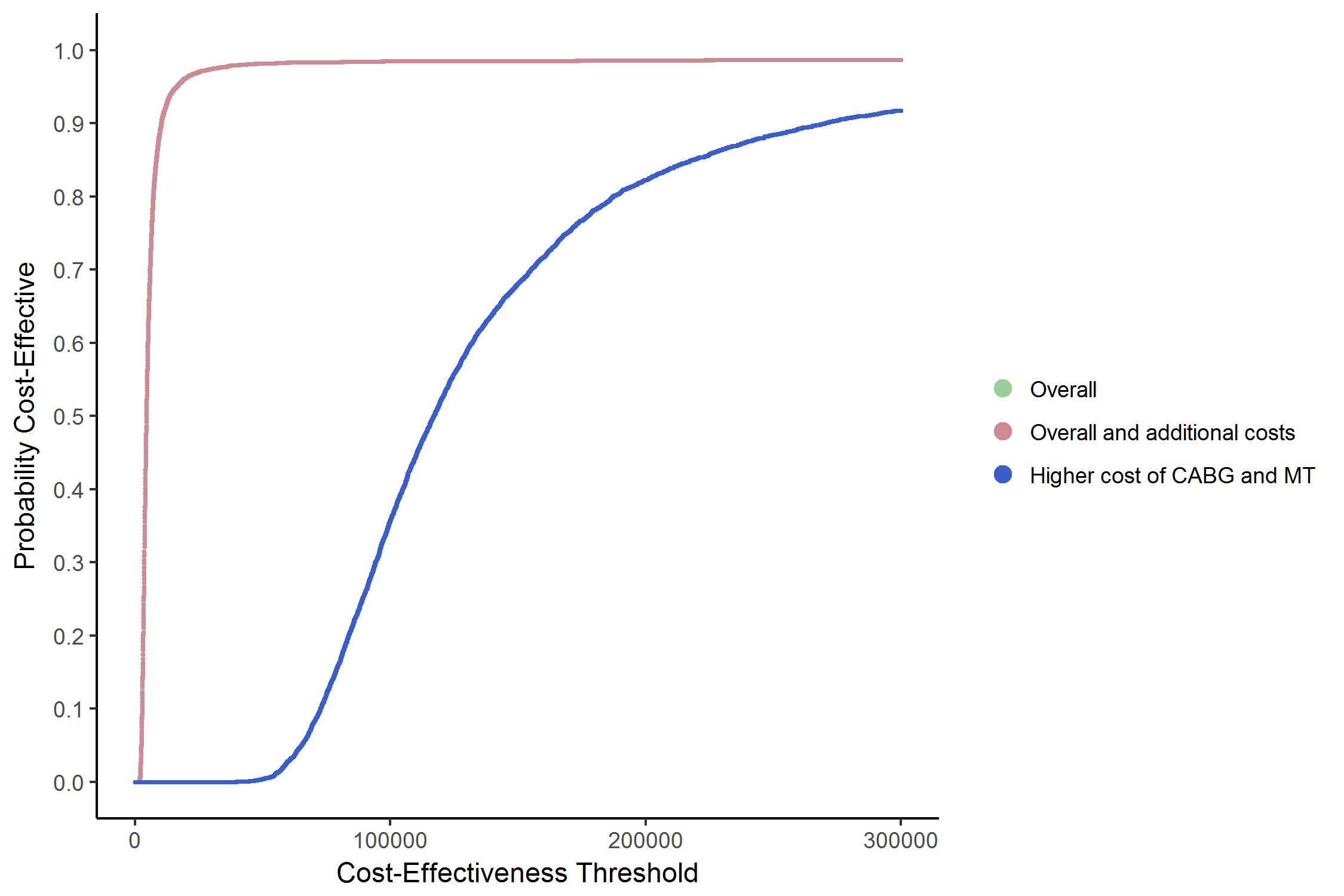


Footnote: In 2 analysis (Overall, Overall and additional costs), the probability that PCI is cost-effective compared to CABG is 62%, even at low threshold values. In Higher Cost analyses, the probability that PCI is cost-effective compared to CABG is lower than 40% at threshold up to 100,000 US dollars.

PCI percutaneous coronary intervention, CABG coronary artery bypass grafting, threshold is in US dollars.

Figure S3. Cost-effectiveness acceptability curves of Coronary Artery Bypass Grafting versus Medical Treatment in 3 distinct analysis

#
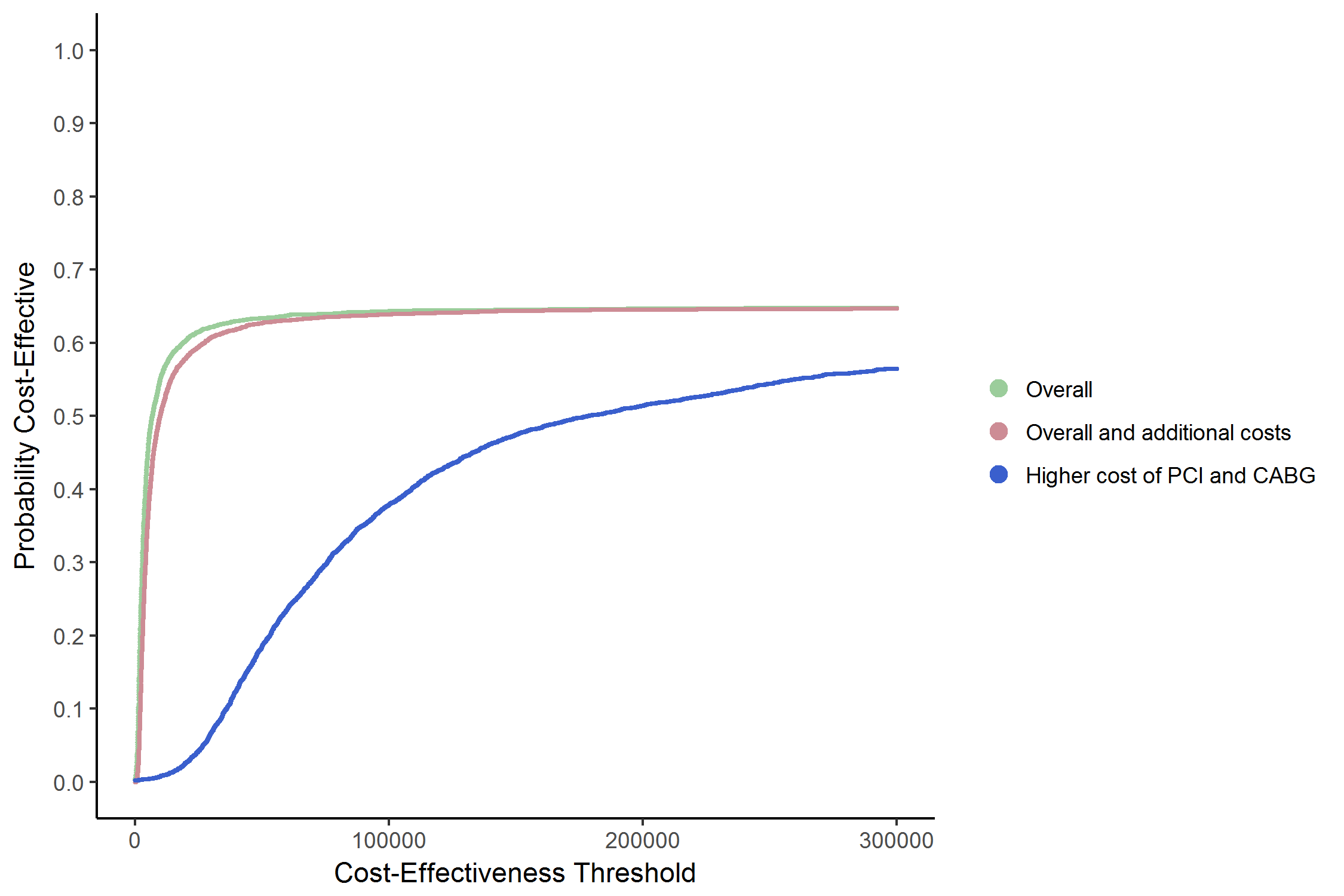


Footnote: In 2 analysis (Overall, Overall and additional costs), the probability that CABG is cost-effective compared to MT is very high, even at low threshold values. In Higher Cost analysis, the probability that CABG is cost-effective compared to MT increases as cost-effectiveness threshold rises to more than 100,000 US dollars.

CABG coronary artery bypass grafting, MT medical treatment, threshold is in US dollars.
